# Methodologies and Challenges for CRISPR/Cas9 Mediated Genome Editing of the Mammalian Brain

**DOI:** 10.3389/fgeed.2020.602970

**Published:** 2020-11-30

**Authors:** Hirofumi Nishizono, Ryohei Yasuda, Tal Laviv

**Affiliations:** Department of Neuronal Signal Transduction, Max Planck Florida Institute for Neuroscience, Jupiter, FL, United States

**Keywords:** HITI (homology-independent targeted integration), SLENDR (single-cell labeling of endogenous proteins with homology-directed repair), iGONAD (improved-genome editing via oviductal nucleic acids delivery), HDR (homology-directed repair), NHEJ (non-homologous end joining)

## Abstract

Neurons and glia are highly polarized cells with extensive subcellular structures extending over large distances from their cell bodies. Previous research has revealed elaborate protein signaling complexes localized within intracellular compartments. Thus, exploring the function and the localization of endogenous proteins is vital to understanding the precise molecular mechanisms underlying the synapse, cellular, and circuit function. Recent advances in CRISPR/Cas9-based genome editing techniques have allowed researchers to rapidly develop transgenic animal models and perform single-cell level genome editing in the mammalian brain. Here, we introduce and comprehensively review the latest techniques for genome-editing in whole animals using fertilized eggs and methods for gene editing in specific neuronal populations in the adult or developing mammalian brain. Finally, we describe the advantages and disadvantages of each technique, as well as the challenges that lie ahead to advance the generation of methodologies for genome editing in the brain using the current CRISPR/Cas9 system.

## Introduction

The development of the Cre/Lox technology and its utilization for gene targeting in embryonic stem (ES) cells in mice in the 1990's has allowed researchers to develop genetically modified mice. This technology has provided the means to address the link between genes, neuronal function, and behavior (Silva et al., 1992; Tsien et al., 1996). The subsequent development of numerous transgenic mice has generated a wealth of data, which provides the neuronal protein landscape and its contribution to the function and the development of the mammalian brain (Tsien, 2016). This approach has proven powerful and contributed to a detailed understanding of the molecular basis underlying various brain circuits and behaviors. However, the implementation and investigation of individual genes and proteins in the brain remains time and resource consuming. This is partly due to the necessity of a specialized ES facility and numerous cross-breeding required to minimize genetic heterogeneity. Furthermore, since these genetic manipulation techniques modify all brain cells, analyses of protein function at the single-cell level have been challenging. In particular, the dense environment of the brain undermines the capability to determine the subcellular localization by fusing fluorescent proteins (FPs) or epitope tags to endogenous proteins.

Over the past decade, the emergence and accelerating improvements of clustered regularly interspaced short palindromic repeats/Cas9 (CRISPR/Cas9) technology have led to various applications to rapidly generate transgenic mice models as well as to perform *in vivo* gene editing. This technique has greatly simplified genome editing at the mammalian brain at the level of whole animals and even that of single cells. These developments hold promise to significantly enhance our understanding of the link between protein signaling, brain function, and animal behavior. Here we will discuss current approaches for the generation of transgenic mice with interventions at early embryo development and fertilization stages and strategies for *in vivo* single-cell genetic modification in the brain.

## Rapid Generation of Transgenic Mice

Genetically engineered animal models are an invaluable tool for understanding gene function and disease mechanisms in the brain. When combined with the Cre-LoxP system and viral vectors, they allow spatial and temporal regulation of specific gene function for further investigation. There are two types of genetically-modified animal models: transgenic models, in which a foreign gene together with a promoter is introduced into a safe harbor region such as Rosa26 (Zambrowicz et al., 1997), tightly regulated (TIGRE) genomic locus (Zeng et al., 2008), or at a random location on a chromosome; and gene-targeted models, in which an endogenous gene is deleted (knockout) or altered (knockin). Transgenic models are often used in Cre lines, in which Cre recombinase is expressed in specific cell types via cell-type-specific promoters (Tanahira et al., 2009; Taniguchi et al., 2011; Peng et al., 2013). Gene targeting models are also used in reporter gene expression lines and conditional floxed mice, as well as simple knockout lines (Navabpour et al., 2020). Traditionally, these mouse models have been generated by introducing foreign genes directly into the anterior nucleus of a fertilized egg using micro-glass needles or generating chimeric mice using ES cells (Capecchi, 2005). Many genetically engineered animal models generated by these methods are available at mouse resource banks such as Jackson Laboratory, The European Mouse Mutant Archive (EMMA), the Mutant Mouse Resource and Research Centers (MMRRC), and RIKEN BioResource Research Center (BRC) using the International Mouse Strain Resource (IMSR) online database (Davisson, 2006; Eppig et al., 2015).

**Figure 1:**
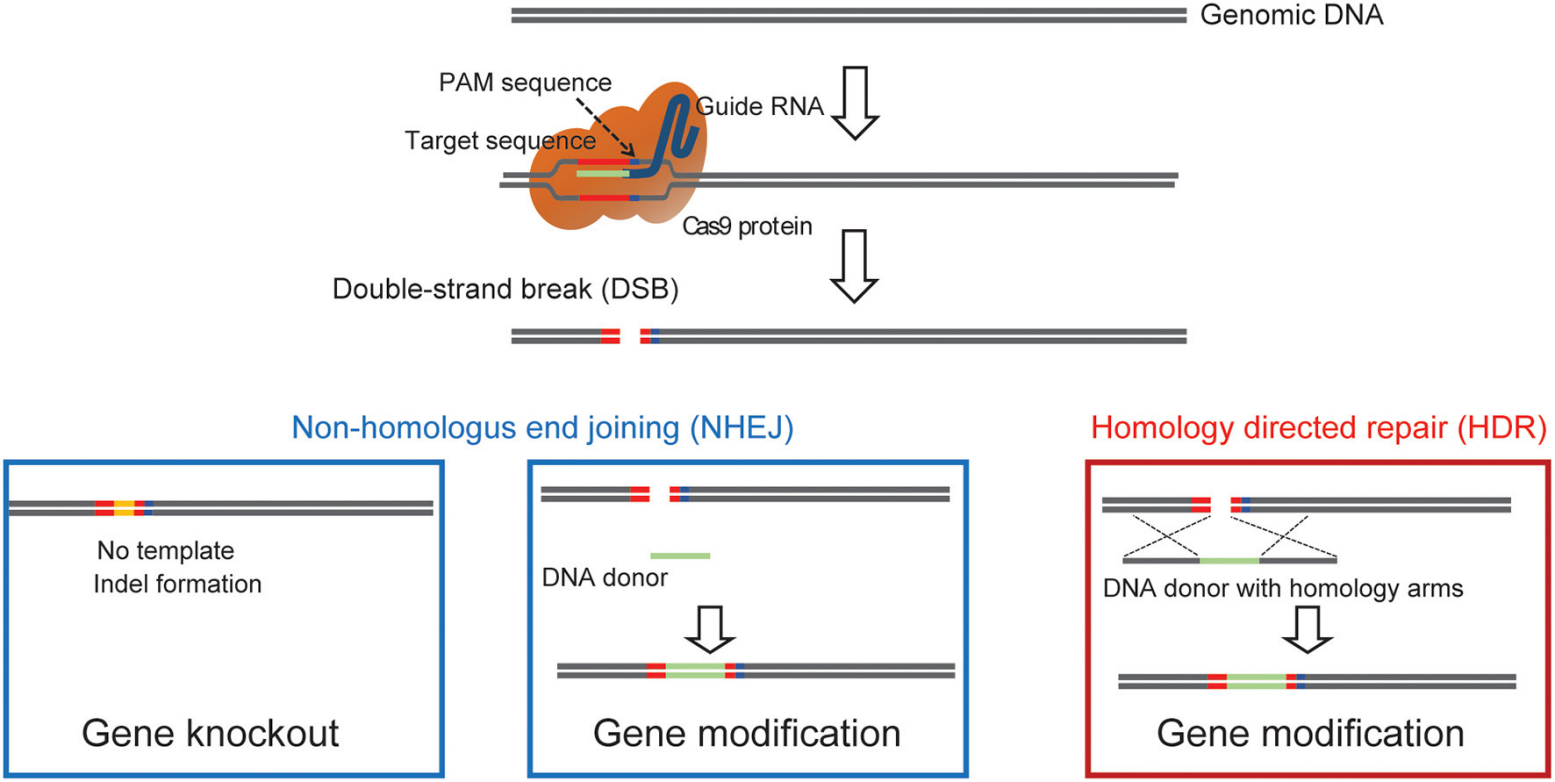
A diagram showing how Cas9 can achieve precise double strand genomic DNA break. The diagram illustrates the use of Cas9 double-strand break to induce gene, NHEJ mediated knockin to label endogenous proteins, and HDR mediated precise knock-in of endogenous proteins.

**Figure 2:**
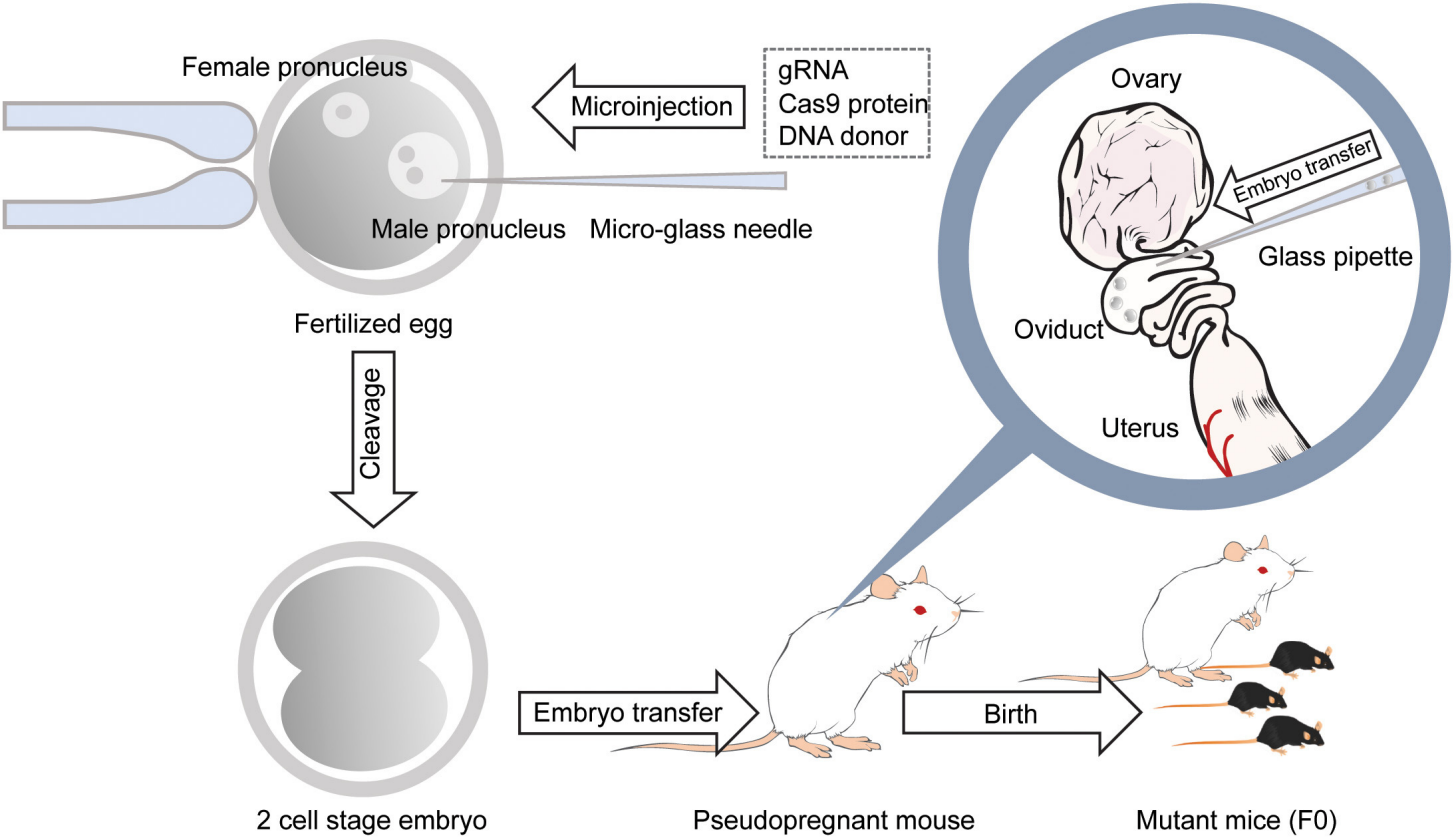
Generation of gene-modified mice using CRISPR/Cas9 by microinjection. With a micro-glass needle, gRNA and Cas9 protein are injected into the male pronucleus of mouse embryos. DNA donors are also injected into the pronucleus when generating knockin mice. Embryos developed at the 2-cell stage the next day are transferred into the oviducts of the pseudopregnant female mouse, and 18–20 days later, F0 mice are born.

One main drawback with the generation of chimeric mice using ES cells is the necessity for an extended period of backcrossing to the target genetic background, such as the C57BL/6 strain. This step is required because, in most cases, the coat color of the mouse, from which injected ES cells are derived, are chosen to be different from that of the host embryo for easy identification of mice with the transgene. Thus, the generated chimeric mice have a mixed background (Carstea, 2009). Recent utilization of the CRISPR/Cas9 system has permitted researchers to develop methods for direct genome editing in fertilized eggs of animals within the target genetic background, saving considerable time for backcrossing (Mashiko et al., 2013; Yang et al., 2013). Cas9 protein binds to the target sequence in the presence of gRNA. It induces double-strand breaks (DSBs), resulting in error-prone non-homologous end joining (NHEJ) or high-fidelity homology-directed repair (HDR) (Figure 1). NHEJ leads to insertions and deletions (indels) and has been used to generate knockout mice. HDR has been utilized to create knockin mice in the presence of a repair template containing homologous sequences (Singh et al., 2015). Thus, knockout and knockin mice can be generated by directly introducing gRNA and Cas9 (protein, mRNA or DNA vector) into the pronucleus of fertilized mouse eggs (Mashiko et al., 2013; Yang et al., 2013) (Figure 2). It takes only 1–2 months to generate F0 mice using this technique. Thus, this method provides a much faster turn-around time than that of ES cell-based techniques, which often require more than 1 year. This technique has also enabled researchers to use genetically modified mice in the F0 generation or with fewer crosses to perform phenotypic analysis of whole-brain anatomy and physiology and conduct behavioral assays within a short time (a few months) (Sunagawa et al., 2016; Tatsuki et al., 2016; Miyasaka et al., 2018). The generation of genetically engineered animals by introducing gRNA and Cas9 protein into fertilized eggs has already been achieved in many different species of mammals, including ferrets (Yu et al., 2019), goats (Ni et al., 2014), pigs (Petersen et al., 2016) and non-human primates (Kang et al., 2019), as well as mice and rats (Shao et al., 2014). Recently it has been found that CRISPR complexes can be directly injected into the pronucleus of a fertilized egg using Au nanowire injector (Park et al., 2017). This method may improve the efficiency of the generation of transgenic mice by reducing the physical destruction of fertilized eggs.

**Figure 3:**
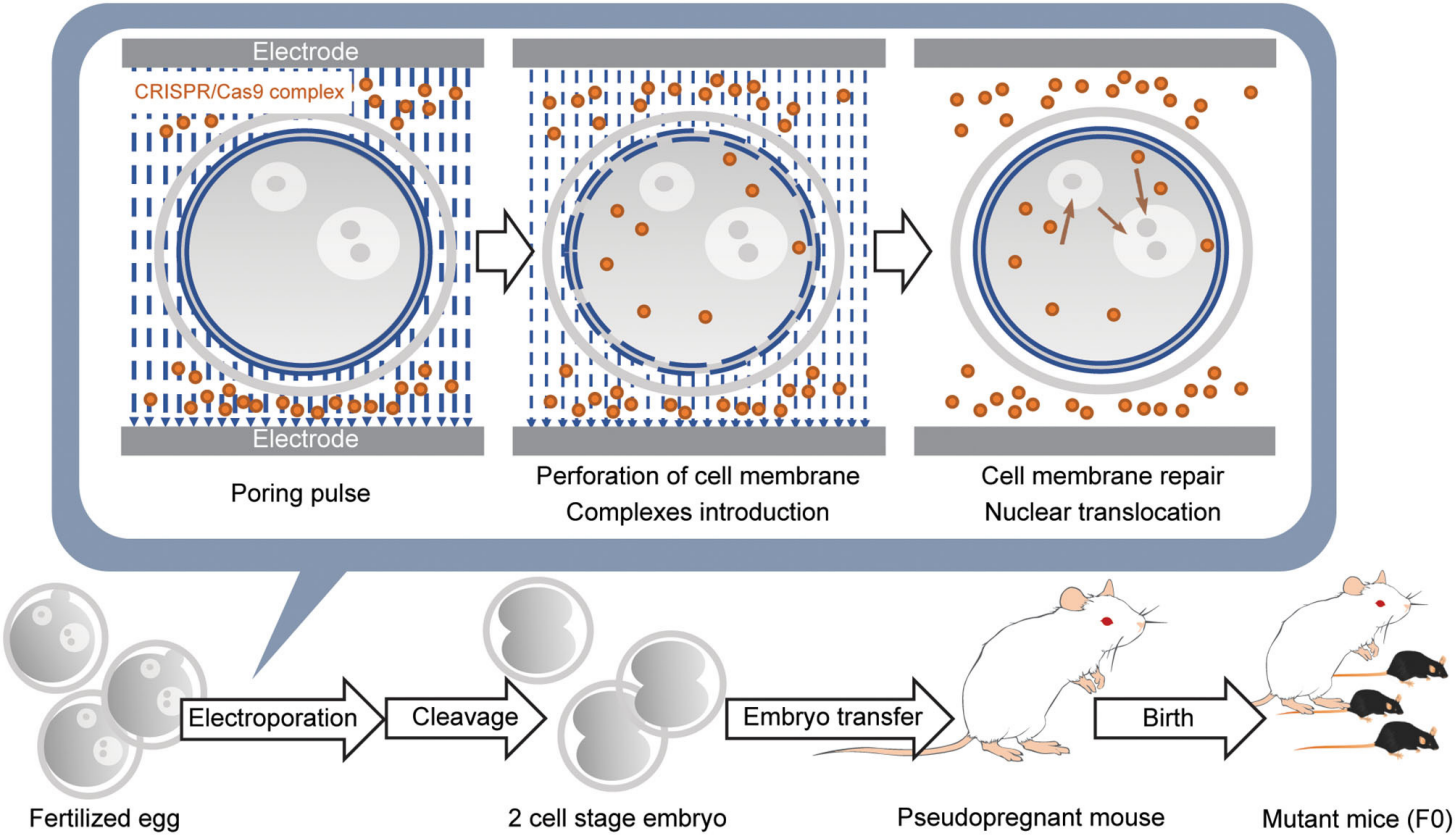
Introduction of CRISPR/Cas9 complex into fertilized eggs by electroporation and generation of the gene-modified mouse. Fertilized eggs are placed in an electroporation buffer containing CRISPR/Cas9 complex filled inside a customized electrode and pulsed to generate perforation of the cell membrane. DNA donors are also included when generating knockin mice. Further pulses lead to the introduction of CRISPR/Cas9 complex into the cytoplasm. When the pulsing is stopped, the cell membrane is repaired. Subsequently, the remaining CRISPR/Cas9 complex in the cytoplasm is translocated into the nucleus. Embryos developed at the 2-cell stage the next day are transferred into the oviducts of the pseudopregnant female mouse, and 18–20 days later, F0 mice are born.

**Figure 4:**
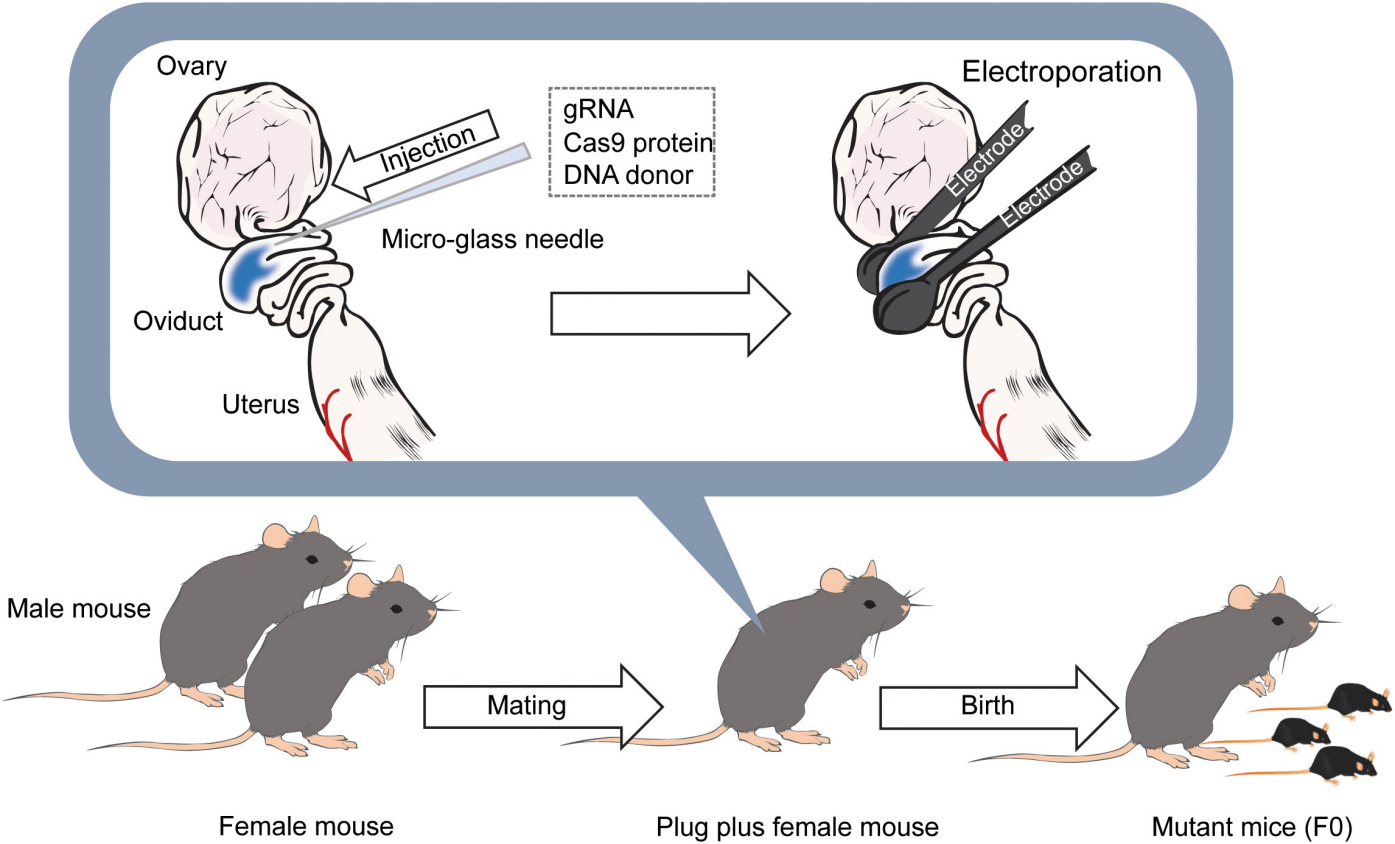
Diagram of the iGONAD method. The day after the mating, gRNA and Cas9 protein is injected into the ampulla of the oviducts of female mice with confirmed plugs using a micro-glass needle. DNA donors are also injected into the ampulla when generating knockin mice. After that, electrodes are placed between the oviducts, and pulses are applied. After 18–20 days later, F0 mice are born.

While direct genome editing of fertilized eggs provides significant advantages in speed, this method requires microinjection, one of the most demanding techniques, as is the case for ES cell-based transgenic mouse generation (Figure 2). In order to simplify genome-editing, a method to introduce gRNA and Cas9 mRNA into fertilized eggs by electroporation has been developed (Kaneko et al., 2014). The efficiency of this approach has been further optimized by using Cas9 protein instead of mRNA (Figure 3) (Hashimoto and Takemoto, 2015; Qin et al., 2015). The use of frozen fertilized eggs instead of fresh ones has further simplified the whole process (Darwish et al., 2019; Nishizono et al., 2020a,b). The efficiency has been reported to be 50–70% for knockin, and close to 100% for knockout (Hashimoto and Takemoto, 2015; Darwish et al., 2019; Gurumurthy et al., 2019b). These improvements hold great promise for a rapid generation of transgenic mouse models, which will allow behavioral and functional screening at the level of F0 mice, and thus circumvent the need for long periods of cross-breeding. The genome editing of fertilized eggs by electroporation has been performed not only in mice but also in rats (Kaneko, 2017) and pigs (Tanihara et al., 2016, 2020). In particular, it has been reported that the electroporation method for editing the genome of porcine fertilized eggs has several advantages over the microinjection method, such as shorter production time and reduced pre- and postnatal death rates (Tanihara et al., 2016). The electroporation-based genome editing method was further simplified by performing genome editing directly on fertilized eggs in the oviduct (Ohtsuka et al., 2018; Gurumurthy et al., 2019b). This technique, called improved-genome editing via oviductal nucleic acids delivery (iGONAD), was achieved by injecting gRNA and Cas9 protein into the oviduct and apply electroporation voltage through a pair of electrical pads (Figure 4).

One prominent drawback of electroporation-based genome editing is that a large donor DNA (over 1 kb) does not reach the nucleus and thus is not integrated into the genome (Hashimoto and Takemoto, 2015). This limitation makes it difficult to generate, for example, conditional knockout (floxed lines) or knockin mice with a long reporter gene (such as GFP). Several approaches have been proposed to solve this problem (Erwood and Gu, 2020). Two research groups have independently developed a method to generate floxed mice by sequential introduction of 5' and 3' LoxP sequences into the 1-cell and 2-cell stages by electroporation (Horii et al., 2017; Liu et al., 2019). These studies demonstrate the feasibility of electroporation methods to generate floxed mice without using microinjection or ES cells. However, the generation of floxed mice by two consecutive electroporations is less efficient than the technique using long transgene and micro-injection (Gurumurthy et al., 2019a). This problem was partially resolved by an improved method called Easi-CRISPR (Quadros et al., 2017), which uses two gRNAs and a long transgene (Vevea and Chapman, 2020). It should be noted that sequential electroporation without a long transgene increases the probability of off-target modifications because each target is associated with different off-target sites.

**Figure 5:**
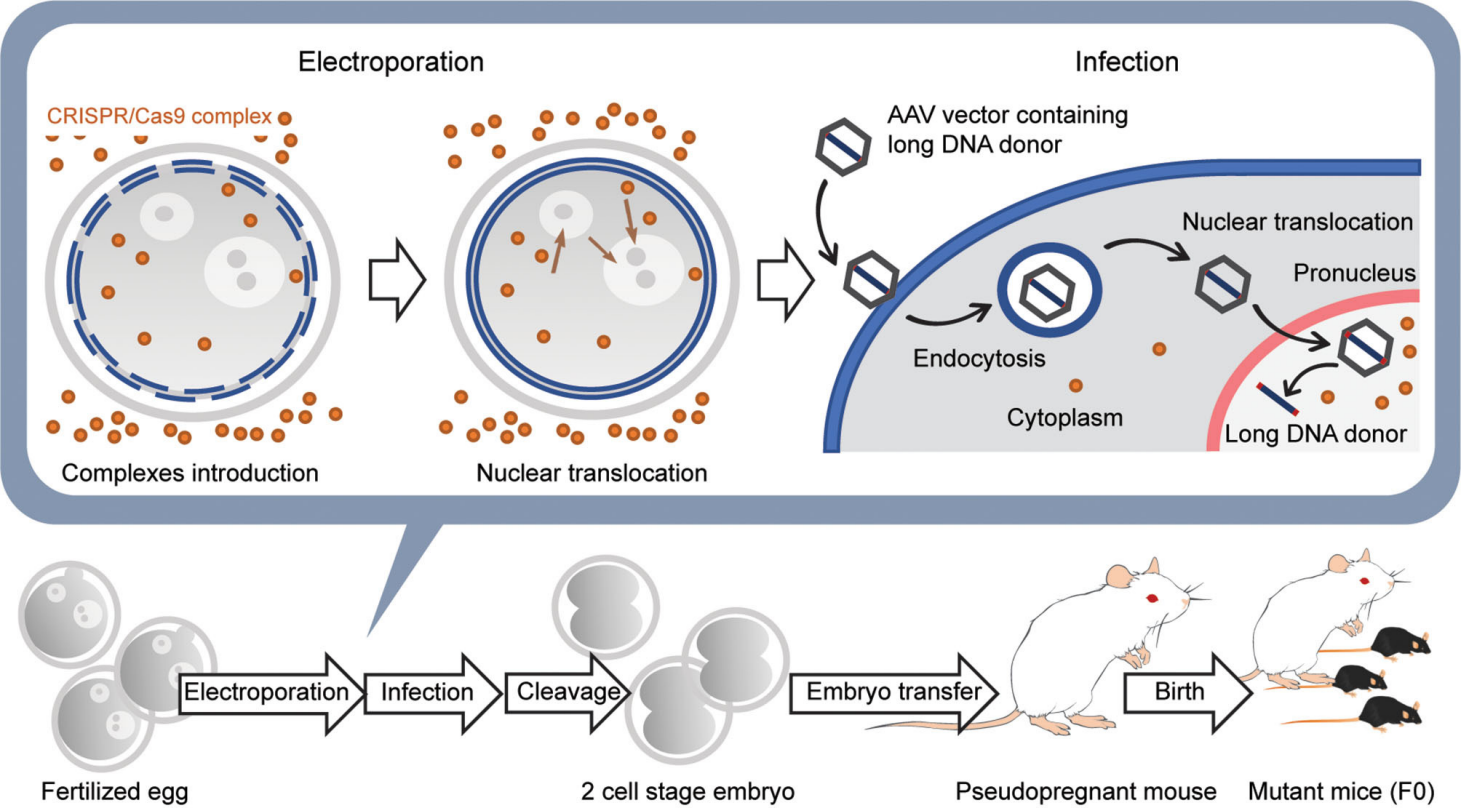
Generation of knockin mice with a long transgene using AAV viral vectors. First, CRISPR/Cas9 complexes are introduced into the fertilized egg using electroporation without long DNA donors. Next, the embryos are moved to the medium containing the AAV vector with the long DNA donor and infected with the viral vectors. The AAV vectors are introduced into the cytoplasm by endocytosis and then translocated into the nucleus using several pathways. The long DNA donors contained in AAV vectors are released in the nucleus and are used for HDR together with the CRISPR/Cas9 complex that was previously transferred into the nucleus. Embryos developed at the 2-cell stage the next day are transferred into the oviducts of the pseudopregnant female mouse, and 18–20 days later, F0 mice are born.

More recently, new techniques based on adeno associated virus (AAV) have been developed to introduce a long DNA donor up to 4.9 kb into mouse fertilized eggs for HDR (Mizuno et al., 2018; Yoon et al., 2018; Chen et al., 2019) (Figure 5). The advantage of using AAV is that the AAV-packaged donor DNA is predicted to be more efficiently translocated into the nucleus than freely diffusing naked DNA. This is due to the efficient translocation of DNA packaged in AAV from the cytosol to the nucleus (Ding et al., 2005). In addition, several small molecules have been reported to increase the efficiency of HDR in genome editing of fertilized eggs (Chu et al., 2015; Riesenberg and Maricic, 2018; Zhang et al., 2020). These methods have enabled the introduction of large reporter genes such as GFP and Cre genes into any location of the endogenous gene, including, for example, a safe harbor like Rosa26. These current methods have made the generation of genetically modified mice using CRISPR/Cas9 genome editing faster and more efficient and should further accelerate the use of genetically modified mice in neuroscience.

## *In vivo* Approaches for Genome Editing in the Brain

Recent advancements in the development and implementation of CRISPR/Cas9 technology has enabled researchers to perform *in vivo* genome editing in the mammalian brain. These methodologies allow rapid and precise knockin, knockout, activation, and inhibition of target genes in a subset of cells, which was previously not technically feasible.

### Protein Knockout Using CRISPR/Cas9

The specific disruption of target genes in the brain has been relatively straightforward to accomplish due to the high efficiency and prevalence of the NHEJ pathway following Cas9-mediated double-strand break. This can be achieved by overexpression of spCas9 and a gRNA sequence, which targets a region in the open reading frame of a gene of interest (Figure 1). It has been demonstrated that the transient expression of Cas9/gRNA in neuronal cultures and brain slices or *in vivo* by plasmid DNA encoding Cas9 and gRNA via virus or *in utero* electroporation (IUE) abolishes the protein from targeted cells (Incontro et al., 2014; Straub et al., 2014). In addition, CRISPR/Cas9 mediated knockout was recently used in ferrets using IUE, and allowed to examine the role of specific proteins for brain development (Shinmyo et al., 2017; Kostic et al., 2019). Alternatively, AAV encoding all CRISPR machinery (Swiech et al., 2015) or that encoding gRNA combined with Cas9 transgenic mice (Platt et al., 2014) were used to transduce a large population of cells in various brain regions to achieve widespread knockout of genes of interest. Due to the large size of spCas9 protein, an AAV based approach is limited and presents some difficulties (see section Discussion). Another main limiting factor is that the “gold standard” for validation of gene perturbation ultimately relies on either immunohistochemistry-based analysis using antibodies (Swiech et al., 2015) or electrophysiological assessment in the case of ion channels and receptors (Incontro et al., 2014; Straub et al., 2014). However, immunohistochemistry relies on the availability of suitable antibodies and may not detect partial protein deletions. Phenotypic analysis of protein knockout for functional or structural markers can only distinguish severe phenotypes and is not suitable for every protein target. Previous work has examined the knockout efficiency in single cells using laser microdissection followed by post-*hoc* sequencing of the target site (Steinecke et al., 2019). Future development of high throughput approaches for single-cell genotyping is required to overcome these limitations and to increase the throughput of CRISPR/Cas9 protein knockout in the brain.

### Gene-Specific Activation and Inhibition Using CRISPR-Cas9

The use of CRISPR/Cas9 to inactivate genes in the brain using directed double-strand DNA cut can lead to the expression of truncated proteins, which can potentially affect protein function. This may impose additional heterogeneity, on top of that induced by imprecise NHEJ repair, in phenotypes in a given neuronal population. To solve some of these issues, an engineered variant of a dead Cas9 (dCas9), which lacks nuclease activity and thus simply binds to specific genomic sites, was developed to modulate gene function via transcriptional control (Qi et al., 2013). To further direct the gene-specific regulation, researchers have developed CRISPRi, in which dCas9 is fused to the transcriptional inhibitor KRAB and targeted to a specific gene near its transcriptional start site in *Escherichia coli* (Gilbert et al., 2014). CRISPRi was later implemented in the mammalian brain (Zheng et al., 2018). The authors utilized CRISPRi to reduce neurotransmitter release using transcriptional inhibition of 4 different genes that control vesicle release in dissociated neurons, with high efficiency and specificity. Due to the size constraints of Cas9 and the KRAB fusion, the authors utilized Lentiviruses (LV) to express CRISPRi in the hippocampus. By manipulating excitatory or inhibitory neurons in the mouse dentate gyrus (DG) using different LV promoters, the authors demonstrated differential effects of neuronal silencing on the excitatory-inhibitory balance during spatial learning. The high efficiency of the CRISPRi platform and its delivery via LV allow researchers to achieve combinatorial gene knockdown. A recent report (Tian et al., 2019) utilized this property to conduct a large-scale CRISPRi-based screening on neurons derived from human induced pluripotent stem cells (iPSC) and uncovered different gene targets specific for neuronal survival and development.

As for gene activation, the CRISPR activation (CRISPRa) system utilizes a VP64-p65-Rta (VPR) based transcriptional enhancer fused to dCas9 and was recently established for enhancing specific neuronal gene expression (Savell et al., 2019). This allowed for the efficient targeting and multiplexed activation of numerous genes across different neuronal populations. This system recently allowed to mimic gene programs in the reward circuitry following exposure to drugs of abuse. The complex transcriptional responses of the dopaminergic system have been analyzed by characterizing the effects of CRISPRa-mediated gene activation on cell-specific responses and physiological phenotypes (Savell et al., 2020). One potential drawback for CRISPRa is that induced gene activation levels may differ from that induced by endogenous transcriptional elements. Future improvements will be necessary to achieve quantitative modular control of gene activation using CRISPRa. The recent development of CRISPRa transgenic mice enabled researchers to increase gene expression of two genomic targets in the hypothalamus and correct haploinsufficiency related to obesity and abnormal metabolic disease state (Matharu et al., 2019). CRISPRa transgenic mice were also used to induce the conversion of adult astrocytes to neurons by multiplex activation of genes in the mouse brain (Zhou et al., 2018).

Another recent adaptation of CRISPR/Cas9 includes the development of base editors (BE), which enable researchers to correct or alter single base pairs at desired genomic locations (Gaudelli et al., 2017). BE is made of dCas9 or nickase fused to an enzyme that converts a base to another. BEs for C to T and G to A have been developed (Komor et al., 2016). BEs were further optimized to modulate specific base pairs within the neuronal genome in the adult mouse brain using a split-BE dual AAV approach, which is necessary to accommodate their relatively large size (Levy et al., 2020). These approaches could be used in the future for engineering genetic models for neuronal disorders with known point mutations or for targeting and developing a therapeutic intervention for disorders.

### Protein-Specific Labeling Using CRISPR-Cas9 Engineering

In order to understand protein function in the brain, it is crucial to examine the subcellular localization and the dynamics of signaling proteins. Tagging endogenous proteins with fluorescent proteins at the level of the whole organism/brain has been challenging and can result in adverse disruptions at the level of gene and protein expression (Fortin et al., 2014). In addition, the use of antibodies for analysis of protein localization depends on their sensitivity and specificity and does not allow for high-resolution subcellular localization due to the high cellular density of brain tissue. To overcome these limitations, several methodologies that enable cell-specific labeling of endogenous proteins in the brain using CRISPR-Cas9 have been developed (Figure 1). The techniques can be generally divided into two classes, one using HDR-based gene editing and the other using NHEJ mediated recombination.

### HDR Based Methods

**Figure 6:**
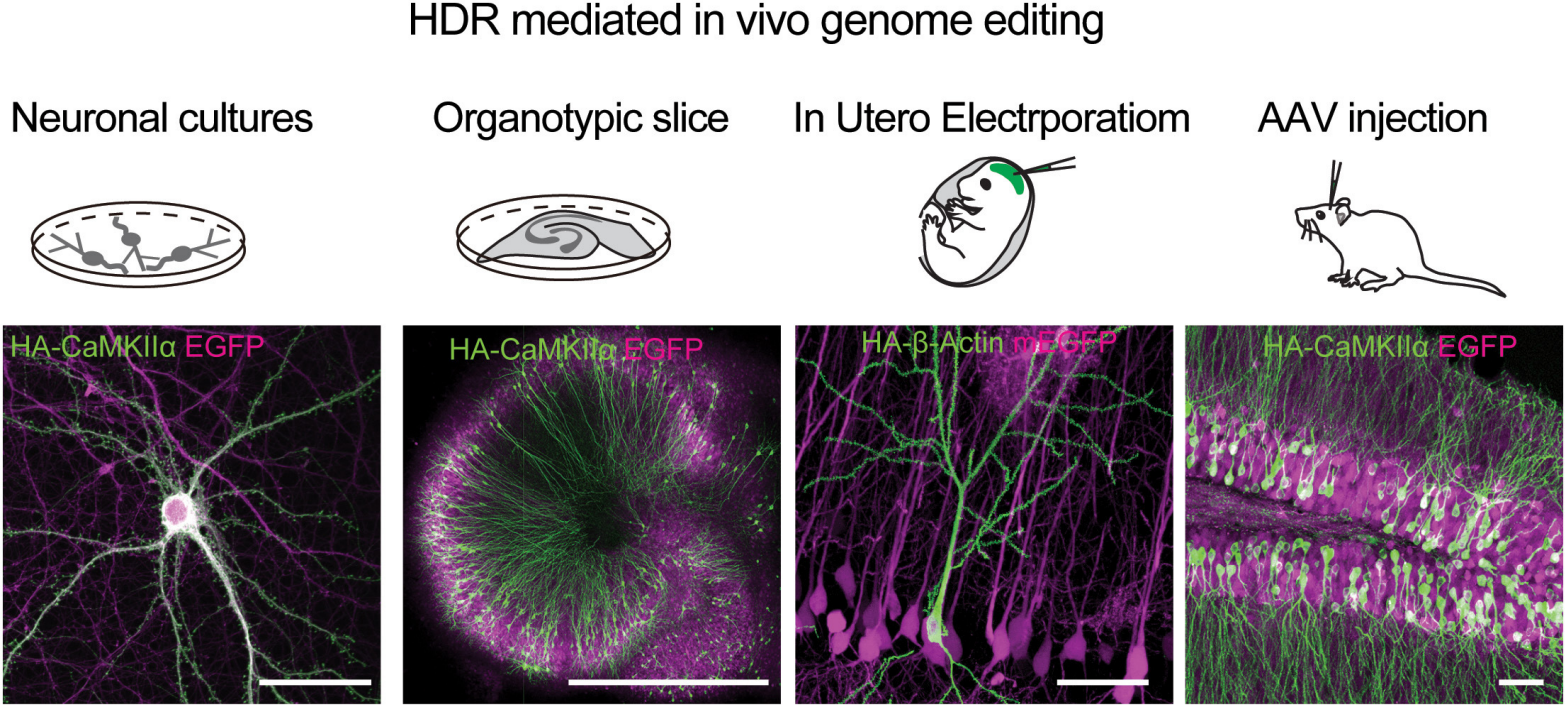
HDR mediated genome editing using SLENDER to label endogenos proteins in the brain. Examples of using vSLENDER in neuronal dissociated cultures (HA-CaMKII-alpha), organotypic slice cultures (HA-CaMKII-alpha), SLENDER using *in utero* electroporation, (HA-bActin), and AAV injections in the adult mouse brain (HA-CaMKII-alpha). Scale bars are 50 μm, 1 mm, 50 μm, and 50 μm, respectively. Images reproduced with permission from Mikuni et al. (2016) and Nishiyama et al. (2017).

HDR-based genome editing allows precise integration to a specific genomic location. To enable HDR based editing of endogenous proteins in the brain, a DNA based HDR template that contains matching homology arms flanking the desired genetic tag is necessary. Following a double-strand break in the target genomic region, the DNA template will be used to precisely introduce the tag via HDR. The specific challenge in genome editing of post-mitotic neurons in the adult brain is that HDR mainly occurs in the S and G2 phases of the cell cycle and rarely in the mature neurons (Heidenreich and Zhang, 2016). Circumventing this problem, a method named SLENDR (single-cell labeling of endogenous proteins with homology-directed repair), demonstrated the feasibility of knockin of specific tags to proteins in the mammalian brain (Mikuni et al., 2016). This was achieved via IUE of Cas9, gRNA, and single-stranded donor oligonucleotides (ssODNs) or a plasmid as DNA templates, performed during early brain development, at the peak neurogenesis and cell division (~E12 for layer 2/3 pyramidal neurons of the neocortex). This allowed the insertion of small, highly immuno-staining-compatible epitope tags such as the HA tag to one of a large variety of gene targets (Figure 6). In addition, this method also allows one to label neuronal proteins with a FP for live imaging of endogenous protein targets. HDR mediated genome engineering is a relatively inefficient process compared to error-prone NHEJ and can therefore integrate the tags to a small, sparse subset of targeted cells (~1–10% of cells targeted, depending on the genomic target). While this is a potential drawback for this methodology, it can be harnessed as an advantage for imaging the target protein with high signal-to-noise in small subcellular neuronal compartments such as dendritic spines and axons. In addition, the HDR process is precise compared to NHEJ and, if the gRNA sequence is targeted outside of the genomic coding regions, it can have minimal effects on the expression of the target protein in cells that did not undergo HDR (Mikuni et al., 2016). While knockin of small tags such as the HA tag is efficient, can be achieved via a short ssODN template, and does not produce non-specific background signal, the insertion of a large FP via a plasmid template often causes a problem of background expression due to innate expression from the template itself (even without a promoter) (Tsunekawa et al., 2016). It has been reported that this type of background can be reduced by including a strong promoter (such as CAG) and stop cassette before the template (Tsunekawa et al., 2016).

The recent development of the vSLENDR technique using AAVs for HDR knock-in template can greatly increase the success rate and the number of cells successfully undergoing knockin even in mature neurons in the adult brain (Nishiyama et al., 2017). However, the background expression still remains an issue for some genes (unpublished). Furthermore, this method necessitates the cloning of large fragments of genomic DNA as homology arms to every protein target and is limited to the size of the genetic tag by AAV packaging capacity. In summary, HDR mediated approach allows for precise error-free genome editing in single neurons in the mammalian brain. While insertion of small immune-reactive tags such as the HA tag is efficient and specific, the insertion of larger fragments such as FP is inefficient and could potentially lead to background expression without genomic integration.

### NHEJ Mediated Knockin Strategies

Alternative approaches for genomic knockin and labeling of endogenous proteins have utilized NHEJ mediated techniques. Following a double-strand break at a desired genomic location, a tag is designed with flanking gRNA target sequences, which are also cut by Cas9, leading to the incorporation of the tag sequence at the break location, similar to a blunt cloning procedure (Figure 1) In the first report of such an approach named homology-independent targeted integration (HITI) (Suzuki et al., 2016), the authors use a strategy to introduce SpCas9, a gRNA targeting sequence, and a donor DNA for a FP, using plasmids or AAVs. The HITI template contains the FP with 2 flanking inverted gRNA sequences identical to the targeted genomic cut location. Thus, upon Cas9 cut at the genomic location and the template, non-homologous dependent integration will lead to FP knockin and, at the sam7e time, alteration of the original gRNA sequences, preventing subsequent cutting following genomic integration.

As NHEJ is an error-prone process, the integration of the insert can be associated with indel formation at both junctions of insertions. In the study reporting HITI (Suzuki et al., 2016), the C-terminus tagging of tubulin led to the successful integration of GFP in neuronal cultures and *in vivo*. However, this induced indel formation at variable efficiencies in integrated cells, according to single-cell DNA sequencing. In addition, while the analysis of indels has been restricted to GFP integrated cells (~10% of transduced cells), indel formation probably occurs in the majority of transduced cells without GFP integration. Therefore, fusing a tag to the N terminal of an endogenous target using this approach could potentially lead to knockout of the gene of interest.

Recently, an additional knockin approach termed intercellular linearized single homology arm donor mediated intron-targeting integration (SATI) was developed based on the original HITI method (Suzuki et al., 2019). This approach utilizes a donor containing one homology arm with an upstream intron, splice acceptor, downstream exon, the 3′UTR, and additional genes such as GFP. This approach allows for the insertion and correction of a single base pair, overcoming one of the limitations of HITI. In addition, targeting an intron for the double-strand break should reduce the risk of the indel formation that leads to gene modifications. Interestingly, this approach was effective in achieving knockin in non-dividing neurons to a degree similar to HITI. However, it predominantly induced an unconventional HDR-like process, termed one-armed HDR (oaHDR), and rarely causes an NHEJ mediated blunt knockin. However, this approach still results in indels at the gRNA cut-site junction. The main advantage of this hybrid approach is its ability to correct small mutations in a targeted exon, as well as the downstream/upstream insertion of additional genetic tags. Its potential limitation is the possible formation of mutagenic indels at one side of the insertion junction.

Another recently developed method is an approach named homology independent universal genome engineering (HiUGE) (Gao et al., 2019), which utilizes an NHEJ based cut and paste approach using AAV expression and provides relatively high throughput. This method is designed as a two-part system: one part, the genetic payload, is cleaved using a highly optimized and universal gRNA sequence distinct from the gene-specific gRNA sequence defined in the second part. This method can potentially be used for high throughput labeling. Since the same payload AAV can be used for all targets, specific protein labeling requires designing of only one component, gRNA specific to the target protein. The throughput of this method has been demonstrated by targeting the HA tag and FPs to a large variety of proteins in the brain. While this approach improves the ease of workflow, the throughput and is implementable across various proteins, this feature also leads to significant specificity and sensitivity issues. The generic payloads are designed with compatibility to open reading frame (ORF) integration, +0, +1, and +2 in regards to target sequences. As the integration of NHEJ knockin is not directed by sequence homology, a significant fraction of cells undergo out-of-frame integration (10–20%). Another recent report employed a very similar technique to label multiple synaptic proteins in neuronal cultures and slices using LV- mediated expression (Willems et al., 2020). The authors generated a large array of vectors for NHEJ mediated knockin of GFP to numerous species of synaptic proteins and demonstrated its utility to structural analysis as well as super-resolution imaging of single synapses. While this approach is useful and easy to use, sequencing results from single cells showed frequent indel formation at both integration junctions. The specificity is highly variable across genomic locations and often can result in gene knockout if the gRNA sequence is within the ORF. Another issue of NHEJ is associated with the lack of specificity in the insertion site, which makes it impossible to insert two different tags to different proteins simultaneously. In contrast, this is straightforward with HDR-based techniques using two different templates (Mikuni et al., 2016).

## Conclusions and Future Improvements

Genome editing of fertilized mouse eggs using electroporation does not require expensive equipment and highly demanding microinjection techniques, enabling one to generate genetically engineered mice in a shorter period than ES cell-based methods (Carstea, 2009). In particular, iGONAD, in which fertilized eggs are genetically edited by *in vivo* electroporation in the oviduct, and the method of knocking in a long transgene using an AAV have simplified the gene manipulation and will be widely used (Mizuno et al., 2018; Yoon et al., 2018; Chen et al., 2019; Gurumurthy et al., 2019a) (Figure 5). Similar techniques will be applied to various mammalian species, such as rats (Kaneko, 2017) and pigs (Tanihara et al., 2016). The development of easy methods to introduce transgene longer than 5 kb and to improve the efficiency of genome editing by electroporation will be desirable in the future.

CRISPR-based genome editing generally has issues in off-target effects and mosaicism. Thus, extreme caution is required when using F0 generation transgenic mice. However, multiple studies have reported that analyses of F0 generation mice can be performed by optimizing genome editing efficiency (Sunagawa et al., 2016; Tatsuki et al., 2016; Miyasaka et al., 2018). For example, phenotypic analyses were performed in F0 mice in which the target region was knocked out using multiple optimized gRNAs (Sunagawa et al., 2016; Tatsuki et al., 2016). More recently, this was further extended to generate brain-specific conditional knockout mice in the F0 generation (Miyasaka et al., 2018). The authors created Cre fertilized eggs by *in vitro* fertilization of sperm from Emx1-Cre homozygous mice, which express Cre protein specifically in cortical neurons and glia, and oocytes from wild-type mice. They then genome-edited the floxed constructs using a pair of optimized gRNAs and a long single-strand DNA (lssDNA). These methods have the advantage of significantly reducing the time by short-cutting the cross-breeding process. Future applications of these methodologies could expand genome editing technology to non-model animals such as monkeys (Kang et al., 2019). Further optimization of these methods to increase efficiency and reduce off-target effects will allow researchers to analyze transgenic mice quickly.

For target point mutations, the use of BEs that performs base substitution without DSBs instead of the conventional Cas9 protein should reduce off-target and mosaicism (Kim et al., 2017; Liang et al., 2017; Liu et al., 2018; Ryu et al., 2018). Similarly, genetically engineered mice can be generated using the recently developed PrimeEditor (PE), which has higher flexibility than the BEs (Liu et al., 2020). PE is a combination of Cas9-nickase and the reverse transcriptase. The technique uses a guide RNA with a desired edit that binds to the target site, called a prime editing guide RNA (pegRNA). When the pegRNA binds to the target site and guides the PE to create a nick on the DNA strand, the edited region of the pegRNA is incorporated into the DNA strand by the reverse transcriptase of the PE. Prime editing allows for more flexible base modification by introducing pre-designed edits via reverse transcription. However, a recent preprint reported (Aida et al., 2020) that although genome editing in fertilized eggs using the PE is highly efficient, it can also cause unwanted mutations. Further improvement of genome editing in fertilized eggs using the PE method will be necessary for the future.

The development of *in vivo* genome editing approaches in the mammalian brain using CRISPR/Cas9 holds great promise for advancing our understanding of the precise roles of individual genes and proteins in neuronal function, plasticity, and development. The choice of the method for *in vivo* genome editing ultimately depends on the experimental needs. HDR-mediated approaches such as SLENDER (Mikuni et al., 2016) allow precise integration and provide a simple workflow for inserting a small epitope tag such as the HA tag (Figure 6). It is relatively limited in efficiency and restricted to integration during early brain development. However, these disadvantages can be mostly overcome by the use of AAV for the transduction of the necessary knockin components (Figure 6) (Nishiyama et al., 2017). Moreover, HDR is precise and allows sequence-specific integration, enabling dual-labeling of tags simultaneously to two distinct genomic targets (Mikuni et al., 2016). Potential drawbacks are for experiments that necessitate inserting an FP or a large genetic payload, since constructing a long template is more time consuming, and the insertion efficiency for a large tag is relatively low. In addition, it is critical to validate and consider the background expression of the template using Cas9 negative control samples.

On the other hand, NHEJ mediated tagging is straightforward and can be easily implemented, particularly when experiments require high throughput tagging to different protein targets. This method has been shown to efficiently knockin FPs and small tags in neuronal cultures and *in vivo* (Figure 6). In addition, there is less restriction on the size of the insert since there is no requirement for genomic homology arms. The disadvantages include the high probability of indel formation induced by the NHEJ mediated process, which may result in unwanted disruption of gene expression in addition to lower specificity and precision of genomic tag. Thus, particular caution is required when attempting to introduce a genetic tag to the N terminus of a protein using NHEJ-based methods. In general, it is best practice to use a gRNA sequence that produces a cutting site near the start or stop codon, but outside of the ORF, to minimize disruption in protein expression. Another drawback is the lack of specificity in integration, limiting the targeting of multiple genetic tags simultaneously (Gao et al., 2019).

Several general points to consider using CRISPR/Cas9 in the brain are Cas9 expression and delivery route, potential cellular phenotypes due to overexpression, and non-specific activity. First, spCas9, the most prevalent and characterized Cas9 variant, is a relatively large genetic payload (4.2 kb), and this poses specific challenges to neuroscience applications. AAVs can achieve widespread neuronal transduction but can only carry a load of 4.7 kb, complicating the AAV-based delivery of Cas9. Possible solutions include the use of short promoters in AAVs (Swiech et al., 2015; Suzuki et al., 2016; Nishiyama et al., 2017), IUE mediated expression of Cas9 (Mikuni et al., 2016; Shinmyo et al., 2016, 2017), and the use of dual-AAV for split Cas9 integration (Chew et al., 2016; Levy et al., 2020). It is also possible to use LV to express Cas9 due to their larger packaging capacity (Savell et al., 2019; Willems et al., 2020). The choice for Cas9 delivery relies on specific experimental needs; for example, the choice between sparse (IUE) or dense labeling (virus) in the brain. Other non-viral applications such as nanoparticles have been continually improving and hold promise for future therapeutic CRISPR/Cas9 applications (Wei et al., 2020). Another potential issue is the possible adverse immunological host response elicited by a high titer of AAV-Cas9 in animals (Chew et al., 2016). Also, a high concentration of Cas9 may cause non-specific nuclease activities, although previous reports did not find evidence for widespread non-specific Cas9 activity (Iyer et al., 2015). It would be a good practice to examine potential non-specific disruptions of genomic sequences and to include Cas9 expressing cells/animals with control gRNA as a negative control group.

In conclusion, rapid advancements in methodologies for gene manipulation and protein tagging in the brain have been advancing our understanding of protein signaling in the brain. Future implementation of these techniques in combination with optogenetic control of protein localization and signaling using photo-sensing domains such as light-oxygen-voltage LOV2 (Yazawa et al., 2009) or cryptochrome 2 (CRY2)-based systems (Kennedy et al., 2010) could lead to spatial and temporal control of endogenous proteins. Furthermore, multiplex tagging of proteins with different FPs could allow for monitoring interactions between endogenous proteins with fluorescence resonant energy transfer (FRET) in live tissues. Finally, implementation of CRISPR/Cas9 genome engineering in different animal models, which have been traditionally precluded from standard genetic engineering, could lead to breakthroughs in genetic manipulations and dissection of neuronal circuits in various mammalian model organisms.

## Author Contributions

All authors listed have made a substantial, direct and intellectual contribution to the work, and approved it for publication.

## Conflict of Interest

The authors declare that the research was conducted in the absence of any commercial or financial relationships that could be construed as a potential conflict of interest.
